# Persistent sciatic artery within the paraneural sheath of the sciatic nerve at the popliteal fossa

**DOI:** 10.1186/s40981-021-00479-z

**Published:** 2021-10-06

**Authors:** Keiko Ogami-Takamura, Hiroaki Murata, Tetsuya Hara

**Affiliations:** 1 Department of Macroscopic Anatomy, Nagasaki University Graduate School of Biomedical Sciences, Nagasaki University, 1-12-4 Sakamoto, Nagasaki, 852-8523 Japan; 2 Department of Anesthesiology and Intensive Care Medicine, Nagasaki University Graduate School of Biomedical Sciences, Nagasaki University, 1-7-1 Sakamoto, Nagasaki, 852-8501 Japan

To the Editor,

Persistent sciatic artery (PSA) is a rare vascular anomaly resulting from incomplete obliteration of the embryonic dorsal axial artery [1, 2]. The prevalence of this phenomenon is estimated to be 0.03 to 0.06%. During embryonic development, the blood flow of the popliteal artery is provided from both the sciatic artery and femoral artery. The sciatic artery normally undergoes gradual and complete involution after the development of the femoral artery. Typically, PSA originates from the internal iliac artery, then coursing along with the sciatic nerve and runs into the popliteal artery [3]. PSA is sometimes accompanied by a hypoplastic femoral artery [1, 4]. We identified an aberrant artery within the paraneural sheath around the bifurcation of the tibial nerve (TN) and the common peroneal nerve (CPN).

When we performed pre-scanning of ultrasound-guided popliteal sciatic nerve block for a 67-year-old female patient who was going to undergo left total knee replacement, a hypoechoic round pulsating structure was identified between TN and CPN within the paraneural sheath at the popliteal fossa (Fig. 1). It turned out to be an artery by using the color Doppler. The arterial structure could be traced back to the mid-thigh along with the sciatic nerve and then it traveled away from the sciatic nerve into the biceps femoris muscle and tapered off. When the arterial structure was chased peripherally, it ran into the popliteal artery. We advanced the block needle into the paraneural sheath at the bifurcation of TN and CPN by using an in-plane technique and carefully avoiding the arterial structure. Then, 20 ml of 0.25% levobupivacaine was incrementally injected. We also performed ultrasound-guided femoral nerve block at the level of the inguinal ligament while visualizing a normal-sized femoral artery and placed a catheter for postoperative continuous femoral nerve block. Postoperative analgesia was excellent, and nerve block-related complications were not observed.

**Fig. 1:**
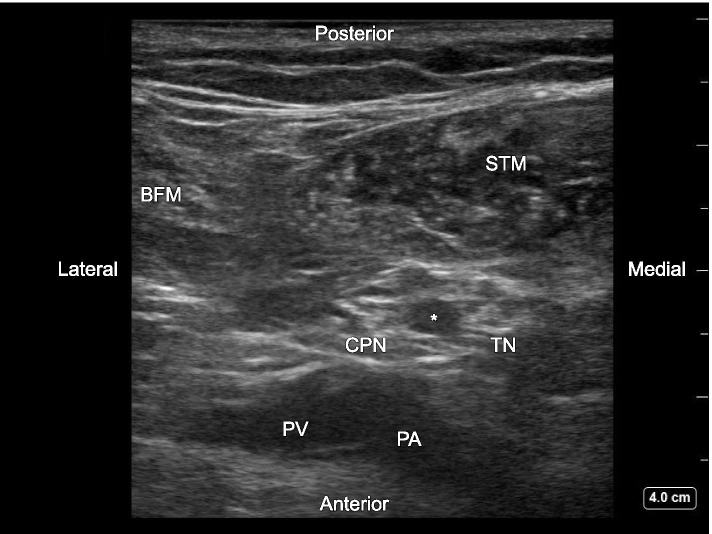
Ultrasound image of an artery within the paraneural sheath between the tibial nerve (TN) and the common peroneal nerve (CPN) at slightly peripheral to the bifurcation of the sciatic nerve. A round hypoechoic pulsating structure (asterisk) was identified between two honeycomb structures (TN and CPN). The diameters of the artery between TN and CPN (asterisk) and the popliteal artery that were measured by the caliper function of the ultrasound machine were 3.2 × 4.2 mm and 5.0 × 6.1 mm, respectively. TN, tibial nerve; CPN, common peroneal nerve; BFM, biceps femoris muscle; STM, semitendinosus muscle; PA, popliteal artery; PV, popliteal vein

In the present case, a presumed PSA was identified within the paraneural sheath at the bifurcation of TN and CPN, which is usually filled with loose connective tissue and is a target of local anesthetic injection during the ultrasound-guided popliteal sciatic nerve block [5]. Considering the anatomical characteristics, the observed arterial structure is presumed as PSA type 4 (Pillet classification modified by Gauffre; incomplete PSA in which its lower part has persisted with a completely developed femoral artery) or ScPi Class II (Ahn-Min’s classification; complete femoral artery and incomplete PSA) [4]. Vascular structures that we need to notice during ultrasound-guided popliteal sciatic nerve block are the popliteal artery and vein outside the paraneural sheath. They may be landmarks to identify the sciatic nerve, but they are rarely injured during the procedure. Although the existence of such aberrant artery would not necessarily result in giving up providing sciatic nerve block, regional anesthesiologists should take care of an artery and a vein locating within the paraneural sheath of the sciatic nerve at the popliteal fossa to avoid unpredictable adverse event caused by their injury, irrespective of the nomenclature or classification of such artery as presented in this report.

## Data Availability

All data generated or analyzed during this study are included in this published article.

## Abbreviations

PSA: Persistent sciatic artery
TN: Tibial nerve
CPN: Common peroneal nerve
